# Ferroptosis as a therapeutic nexus: traditional Chinese medicine interventions in rheumatoid arthritis

**DOI:** 10.3389/fimmu.2026.1768013

**Published:** 2026-02-19

**Authors:** Ruibin Yang, Ziwei Yang, Zhuan Feng, Yaxin Ding, Jiali Yang, Yinuo Zhang, Zhi-Nan Chen, Fei Huo, Jiao Wu

**Affiliations:** 1 Department of Cell Biology, National Translational Science Center for Molecular Medicine, Fourth Military Medical University, Xi’an, China; 2 State Key Laboratory of New Targets Discovery and Drug Development for Major Diseases, Xi’an, China

**Keywords:** combined treatment, ferroptosis, inflammation, rheumatoid arthritis, traditional Chinese medicine

## Abstract

Ferroptosis is an iron-dependent, lipid peroxidation-driven form of programmed cell death that plays an important role in neurodegenerative, neoplastic, and autoimmune diseases. Recent studies in Rheumatoid arthritis (RA) have shown that iron metabolism disorders caused by iron overload and impaired transferrin function lead to the production of reactive oxygen species; ferroptosis-associated pathways, such as dysregulation of the System Xc^-^/GPX4 axis dysregulation, NCOA4-mediated ferritin autophagy, endoplasmic reticulum stress and ferroptosis pathway crosstalk; as well as ferroptosis plays a regulatory role in a variety of immune cells, such as T-cells, B-cells, macrophages, etc., which collectively constitute a complex disease regulatory network in RA. Studies have shown that traditional Chinese medicine (TCM) and TCM therapeutics can alleviate RA-related symptoms and improve the disease progression and prognosis of RA by regulating iron metabolism, activating the Nrf2/HO-1 antioxidant pathway, and removing abnormally proliferating synovial fibroblasts (FLS cells). The aim of this review is to comprehensively summarize the therapeutic potential of TCM for RA using ferroptosis as a therapeutic pathway. The aim is to provide a scientific basis for the clinical application of TCM in RA. In major scientific databases (including PubMed, Web of Science, ScienceDirect, and CNKI, covering literature published up to June 2025). The search strategy combines “Chinese medicine”, “TCM”, “ferroptosis”, and “rheumatoid arthritis”, using Boolean operators (AND, OR). This review systematically elucidates the mechanistic underpinnings through which TCM mitigates RA by modulating ferroptosis pathways. This review highlights the potential that Chinese medicine holds in the treatment of RA. The use of ferroptosis as a therapeutic pivot provides new ideas for the treatment of RA and promotes the integration of Western and Chinese medicine.

## 1 Introduction

Rheumatoid arthritis (RA) is an autoimmune disease characterized by synovial inflammation and progressive joint destruction (1), whose global prevalence is increasing and has become one of the leading causes of disability (2). Pathologically, RA is characterized by synovitis, bursa formation and progressive cartilage/bone destruction, ultimately leading to irreversible joint deformity and dysfunction (3). Current Western medical treatment is based on disease-modifying anti-rheumatic drugs (DMARDs) (4), but there are problems such as insufficient efficacy, drug resistance and adverse reactions, and the clinical outcomes of RA patients are not satisfactory (3). In addition, TCM has a long history in the treatment of RA, which is classified as “Bi Zheng” or “Wang Bi” in Chinese medicine, and is believed to be related to the attack of external evils and the lack of positive energy in the body (5). Studies have found that TCM can alleviate disease progression through multi-targeted modulation of the immune network, inhibition of inflammatory factors such as TNF-α and IL-6 expression (6), and inhibition of synovial hyperplasia (7), and has shown synergistic and toxicity-reducing effects in combination with DMARDs (6).

The therapeutic efficacy of disease-modifying antirheumatic drugs (DMARDs) is limited. While early initiation of DMARD therapy helps reduce structural damage, halt disease progression, and improve physical function (8), low-dose methotrexate—still the primary initial treatment—only benefits 25% to 40% of patients and carries adverse effects such as hepatotoxicity and myelosuppression (9). Due to its slow onset, conventional synthetic DMARDs (csDMARDs) often require short-term glucocorticoid bridging therapy for acute inflammation. Even with the advent of biological DMARDs (bDMARDs), a subset of patients remains non-responsive; for instance, 42.9% of methotrexate-refractory RA patients failed to meet ACR criteria after 12 weeks of adalimumab (10). Beyond efficacy, safety concerns persist: csDMARDs can cause systemic adverse events, limiting their use in the elderly or those with comorbidities (11), while bDMARDs increase the risk of serious infections, malignancies, and cardiovascular events (11, 12). Additionally, some DMARDs require high-dose folic acid supplementation or regular intravenous infusions, leading to poor adherence and imposing economic and psychological burdens. Considering these limitations, traditional Chinese medicine (TCM) offers unique complementary value in RA treatment, including reducing drug resistance, improving microcirculation, synergistically alleviating pain, and mitigating systemic toxic side effects.

Recent studies have found that TCM or polyherbal formulation, such as Elettaria cardamomum, Duhuo Jisheng decoction, etc., by regulating iron metabolism, activating the FLS ferroptosis-related signaling pathway, and inhibiting lipid oxidation and ROS accumulation in chondrocytes (13, 14), can effectively alleviate the symptoms of RA patients and slow down the development of the disease, and the targeting of ferroptosis may become a new and effective means of treating RA.

Ferroptosis is an iron-dependent form of programmed cell death. Iron overload and lipid peroxidation are the core features of ferroptosis in RA patients and animal models (15–17). Ferroptosis activation primarily suppresses the proliferation and migration of rheumatoid arthritis fibroblast-like synoviocytes (RA-FLS) by disrupting intracellular redox homeostasis and modulating inflammatory pathways. Clinical datas show that lipid antioxidant levels in FLSs are higher than those in healthy people, and ferroptosis is reduced in RA patient-derived FLSs compared to healthy individuals, confirming their resistance to ferroptosis (18). Studies have confirmed that ferroptosis disrupts the intracellular redox balance, particularly suppresses the activity of GPX4. For instance, semaphorin 5A levels are significantly elevated in the synovial fluid and synovial tissues of RA patients, activating the PI3K/AKT/mTOR signaling pathway, enhancing GPX4 expression. Conversely, the use of RSL3 to specifically block GPX4 disrupts the intracellular redox balance, significantly reducing RA-FLS proliferation and migration (19). Additionally, TNF signaling upregulates molecules such as SLC7A11, promoting cystine uptake and glutathione biosynthesis, protecting RA-FLS from ferroptosis. The application of the ferroptosis inducer IKE reduces the number of fibroblasts in the synovium in the CIA model and decelerates the progression of arthritis (20). Moreover, curcumin-mediated photodynamic therapy (CUR-PDT) promotes ferroptosis by increasing intracellular ROS, decreasing glutathione levels, inducing mitochondrial shrinkage reduce the proliferative, invasive, and migratory capacities of RA-FLS (21). Ferroptosis can also activate innate immunity, release inflammatory mediators, and trigger systemic inflammatory responses. Upon ferroptosis activation, the levels of proinflammatory cytokines (such as IL-1β and IL-6) released by FLSs change, weakening their autocrine proinflammatory loop and suppressing their invasive behavior (22). Taken together, these studies fully confirm that activation of ferroptosis inhibits the proliferation and migration of RA-FLS and alleviates disease progression. Meanwhile, TCM has significant therapeutic potential in the treatment of RA by modulating cellular ferroptosis.

Although it has been confirmed that TCM regulates ferroptosis to intervene in the development of RA, the related mechanism research is still imperfect, the TCM treatment method for RA is not systematic enough, and the clinical guideline is still dominated by western drugs. However, it cannot be ignored that TCM combined with Western drug therapy treatment can further enhance the therapeutic effect and alleviate the adverse drug reactions, and TCM or combined TCM and Western drug therapy shows a strong therapeutic potential. The aim of this paper is to establish a complete theoretical framework for the regulation of ferroptosis by TCM in the treatment of RA, and to improve the linkage between TCM and the molecular pathway of ferroptosis. And innovatively proposing to map the activation pattern of ferroptosis in specific stages (early synovitis vs. late bone erosion), this review evaluates the feasibility of TCM in the staged treatment of RA and proposes an integrative medicine strategy to achieve precise RA management.

## 2 Methods

A systematic search was conducted in major scientific databases (including PubMed, Web of Science, ScienceDirect and CNKI, covering literature published up to June 2025). The search strategy used a combination of subject terms and free words, and the Chinese search terms included: “Chinese medicine”, “Chinese medicine”, “TCM”, “ferroptosis “, “rheumatoid arthritis”, etc.; English search terms included: “Traditional Chinese Medicine”, “TCM”, “Chinese herbal medicine”, “ferroptosis “, “rheumatoid arthritis”, “RA” and so on. Search terms were constructed using Boolean logical operators (AND, OR), e.g. (“Traditional Chinese medicine” OR TCM OR “Chinese medicine”) AND (ferroptosis) AND (“rheumatoid arthritis” OR RA).

Literature inclusion criteria were 1) the study was on RA; 2) the study involved TCM or polyherbal formulation intervention; 3) the study was related to ferroptosis mechanisms; and 4) cellular, animal or clinical studies. The exclusion criteria included: 1) non-Chinese and English literature; 2) duplicate publications; 3) literature for which full text was not available; and 4) case reports, conference abstracts and review articles.

The quality of literature was assessed using the Cochrane Risk of Bias Assessment Tool for methodological quality assessment of randomized controlled trials, and the SYRCLE Risk of Bias Tool for Animal Experiments to assess the quality of animal experimental studies, with additional assessment of the quality of animal experimental reports through the ARRIVE guidelines. Two investigators independently conducted literature screening, data extraction and quality assessment, and any disagreement was resolved through negotiation or arbitration by a third investigator.

## 3 Ferroptosis at different stages of RA

There are various staging and grading methods for RA based on different criteria, which are closely related to the choice of RA treatment. According to the imaging changes, RA can be simply divided into early RA and late-stage RA. Early RA is the inflammation progression stage, and the X-ray shows osteoporosis of the proximal interphalangeal joints, a slightly blurred joint space, and erosion has not yet appeared; late-stage RA shows obvious bone erosion, worm-eaten-like destruction, significant narrowing of the joint space, and even joint ankylosis. The pathological progression of RA is closely related to the dysregulation of immune cells and mesenchymal cell proliferation. In addition, metabolic changes have a significant impact on immune cell function and tissue damage at different stages of the disease (23). It has been found that RA patients often suffer from disturbed iron metabolism, intracellular Fe2^+^ accumulation, upregulation of lipid oxidation levels and GSH depletion, leading to abnormal proliferation of synovial fibroblasts or chondrocyte ferroptosis, exacerbating the progression of RA, confirming the strong association between RA and ferroptosis. However, it is worth noting that a large body of evidence suggests that ferroptosis may be involved in RA disease regulation at different stages of RA progression through different response mechanisms. In early RA, the intracellular antioxidant system (GPX4/SLC7A11/Nrf2) is relatively intact, and the ferroptosis-resistant system functions partially to protect synovial tissues; in late-stage RA, these defense mechanisms are suppressed by inflammatory signals, and iron metabolism imbalance and lipid peroxidation are exacerbated, resulting in ferroptosis activation and promotion of inflammatory malignancy. activation, which promotes the vicious circle of inflammation and joint destruction. Based on this, further validation of staging and establishment of early-late-stage ferroptosis typing models are important for RA treatment.

### 3.1 Early RA

Early RA manifests mainly as pain in simple joints or fingers, wrists, toes and ankles and progressively worsens with increased disease activity. The pathological process mainly involves disruption of immune tolerance in lymphoid organs, dysregulation of intracellular signaling and impaired differentiation of regulatory T-cells, which not only affects the normal functioning of the immune system, but also promotes inflammatory responses and tissue damage through a variety of mechanisms (23). It has been found that elevated local iron concentrations and increased iron loading have been observed in early RA, and iron is an important regulator of immune responses, and its metabolism is important for autoimmune diseases, including RA (24). Iron overload is not only involved in innate immunity such as regulating macrophage polarization, recruiting neutrophils, and modulating NK cell activity, but also regulates T and B cell-mediated immune responses through adaptive immunity (24). In addition, mitochondrial reactive oxygen species production was increased five-fold in whole blood and monocytes of RA patients compared to healthy individuals (25), and levels of other important lipid peroxidation products (e.g., 8-OHdG, MDA, and 4HNE) were higher in synovial membranes and synovial fluid than in healthy controls (20), while GSH and glutathione peroxidase production decreased, confirming that the oxidative microenvironment has been disrupted (26). Treatment of cells isolated from synovium and synovial fluid of RA patients with the GPX4 inhibitor RSL3 specifically increased cell death in fibroblast-activating protein-alpha (FAPα^+^) fibroblasts but had no effect on cell death in macrophages, endothelial cells, T-cells, or B-cells, whereas low-dose imidazolidinone analogues of erastin (IKE), in combination with the anti-TNF drug etanercept, when used in combination with the anti-TNF drug to treat a mouse model of collagen-induced arthritis, was able to induce ferroptosis in synovial fibroblasts and slowed down the progression of arthritis in a mouse model of CIA (20). Similarly, lipopolysaccharide (LPS)-induced synoviocytes can be used as a cellular model of synovitis, with elevated MDA levels and iron and reduced GPX levels in LPS-induced synoviocytes, while the herbal medicine Icariin (ICA) inhibits through the Xc^-^/GPX4 axis, thereby enhancing cell survival in lipopolysaccharide-induced synoviocytes (27).

In addition, the System Xc^--^GPX4 axis is expressed relatively intact in early RA, possibly counteracting some of the lipid peroxidation and preventing the rapid onset of ferroptosis. Also, ferroptosis resistance systems such as the Nrf2 antioxidant system play an important role in early RA. Enhanced gene levels and expression of Nrf2/HO-1 have been observed in RA patients, exerting its anti-inflammatory and antioxidant effects, and the lack of Nrf2 leads to elevated levels of joint alterations, which can lead to oxidative stress, pro-inflammatory cytokine release, and immune cell recruitment (28). The TCM Resveratrol, Tanshinon and Curcumin resist ferroptosis in RA-associated animal or cellular models by activating the Nrf2 pathway to up-regulate systemic Xc^-^ function, inhibiting ROS and MDA production, suppressing the activation of NF-κB and the proliferation and migration of RA-FLS (29, 30).

### 3.2 Late-stage RA

Late-stage RA is also the highly active phase, in which chronic inflammation gradually erodes the cartilage around the joints, resulting in significant bone destruction, joint deformity, and dysfunction. This phase is characterized by the amplification and maturation of self-reactive lymphocytes that encounter self-antigens in peripheral tissues and form tertiary lymphoid structures. These pathological changes maintain chronic destructive inflammation while compromising the synovial tissue’s homeostasis and tissue repair (23). Disease severity correlates with synovial iron overload and lipid peroxidation levels, including reactive oxygen species (ROS) accumulation, disruption of iron metabolic homeostasis, and lipid peroxidation damage associated with ferroptosis (20). Lipid peroxidation and mitochondrial dysfunction caused by iron overload may promote the abnormal proliferation and fibrosis process of synovial fibroblasts (FLS). Excessive production of reactive oxygen species (ROS) and lipid peroxidation in the synovial microenvironment are the driving factors of synovial fibrosis (31, 32). The level of ferroptosis in RA-FLS is significantly higher than that in osteoarthritis patients, and ferroptosis can enhance its antigenicity by promoting the expression of citrullinated histone H3 (cit-h3), further activating the immune response and exacerbating fibrosis (33). In addition, ferroptosis-induced glutathione metabolic disorder may disrupt the redox balance of synovial cells and accelerate the process of fibrosis. Meanwhile, pro-inflammatory factors such as TNF-α, IL1β, IL6 and other pro-inflammatory factors inhibit system Xc^-^, further weakening the antioxidant system, and combined with the release of DAMPs (e.g., HMGB1) from iron-dead cells to activate NF-κB and NLRP3 inflammatory vesicles, forming a positive feedback and promoting each other, accelerating joint destruction (34). Notably, there are significant differences in iron metabolism and polarization status between M1 and M2 macrophages, and these differences directly affect their sensitivity to ferroptosis (35). It has been found that M2 macrophages enhanced their susceptibility to ferroptosis because their GPX4 proteins were more readily degraded by p62/SQSTM1-mediated autophagy (36). M1 macrophages usually exhibited higher inducible nitric oxide synthase (iNOS) expression and produce higher amounts of nitric oxide (NO), which inhibits lipid peroxidation and thus enhances resistance to ferroptosis (37). And in a study of a mouse CIA model, it was found that in an iron-rich synovial environment of arthritis, although M2 macrophages were more susceptible to ferroptosis than M1 macrophages, HMGB1 released by M2 macrophages interacted with TLR4 on M1 macrophages, triggering activation of STAT3 signaling in M1 macrophages and promoting inflammatory responses. Inhibition of ferroptosis rescues M2 macrophages and alleviates arthritis by inhibiting the HMGB1/TLR4/STAT3 axis in M1 macrophages (31).

In conclusion, ferroptosis in different stages of RA has a complex mechanism involving interactions between iron metabolism, oxidative stress and inflammatory signaling pathways, etc. The establishment of models for early and late ferroptosis typing plays an important role in the search for potential targets for RA therapy.

## 4 Mechanisms of TCM in modulating ferroptosis for RA management

TCM, as a vital component of China’s cultural heritage, has a rich history documented in ancient medical texts such as Huangdi Neijing (The Yellow Emperor’s Inner Canon), dating back to the Warring States period (5th–3rd century BCE). In this work, the mechanism of RA, or “Bi Zheng”, is described as “When wind, cold and damp are mixed, they create blockage (bi).” The work divides “Bi Zheng” into three categories. When wind pathogen predominates, it is termed migratory bi (xing-bi); when cold prevails, painful bi (tong-bi); when dampness dominates, fixed bi (zhuo-bi). Due to the difference of severity in the three evils of wind, cold, and dampness, as well as the different parts of the body that the evils invade and the different body qualities, different illnesses arise. According to TCM theory, each of the five zang-organs has its corresponding shu-stream points, while each of the six fu-organs has its corresponding shu-stream points. while each of the six fu-organs has its he-sea points. Disease manifestations can be detected through observable signs along meridian pathways. Extensive evidence indicates that TCM not only regulates ferroptosis but also induces it through natural components—from single-herb TCM to polyherbal formulation—offering novel therapeutic strategies for RA (5).

In previous studies, TCM clinical trials for RA often relied on pain relief, morning stiffness, and joint swelling as efficacy indicators, lacking a unified assessment standard. Recently, a Core Outcome Set (COS) for TCM treatment of RA was established through systematic reviews and Delphi surveys, encompassing overall disease assessment, physical and chemical indicators, quality of life, TCM syndromes, and adverse events, thus providing a standardized evaluation framework for natural compounds (38). Specnuezhenide (SPN), derived from Ligustrum lucidum, precisely targets KEAP1, restores the osteoclast-osteoblast balance, modulates the KEAP1/NRF2/ROS axis, and attenuates bone destruction in RA (39). Xuetongsu, by targeting IL-23 and inhibiting the IL-23/IL-17/NF-κB inflammatory signaling axis, has emerged as a potential therapeutic target for RA (40). Notably, recent studies emphasize the critical role of metabolism in RA. Fuzi modulates gut microbiota and microbial bile acid metabolism; the microbial metabolite THDCA acts on the TGR5-cAMP-PKA signaling pathway and the NLRP3 inflammasome to alleviate cold-related arthritis (40). Jingfang Granules alleviate ferroptosis induced by lipid peroxidation in RA rats by increasing short-chain fatty acids in feces and serum, activating the Nrf2/HO-1 pathway, and enhancing antioxidant capacity, thus tightly integrating gut microbiota, metabolism, and ferroptosis (41). Additionally, some nano-drug delivery systems loaded with TCM have demonstrated significant therapeutic effects in RA. Prussian blue nanoparticles loaded with Xuetongsu selectively accumulate in M1 macrophages and osteoclast precursors in inflamed joints through surface modification and may indirectly modulate ferroptosis by regulating iron metabolism and lipid peroxidation (42). The following discussion will integrate recent research advancements to explore the potential role of TCM therapies in treating rheumatoid arthritis (RA) through the ferroptosis pathway.

### 4.1 Single-herb TCM

#### 4.1.1 Nrf2/HO-1 signaling pathway

**Figure 1:**
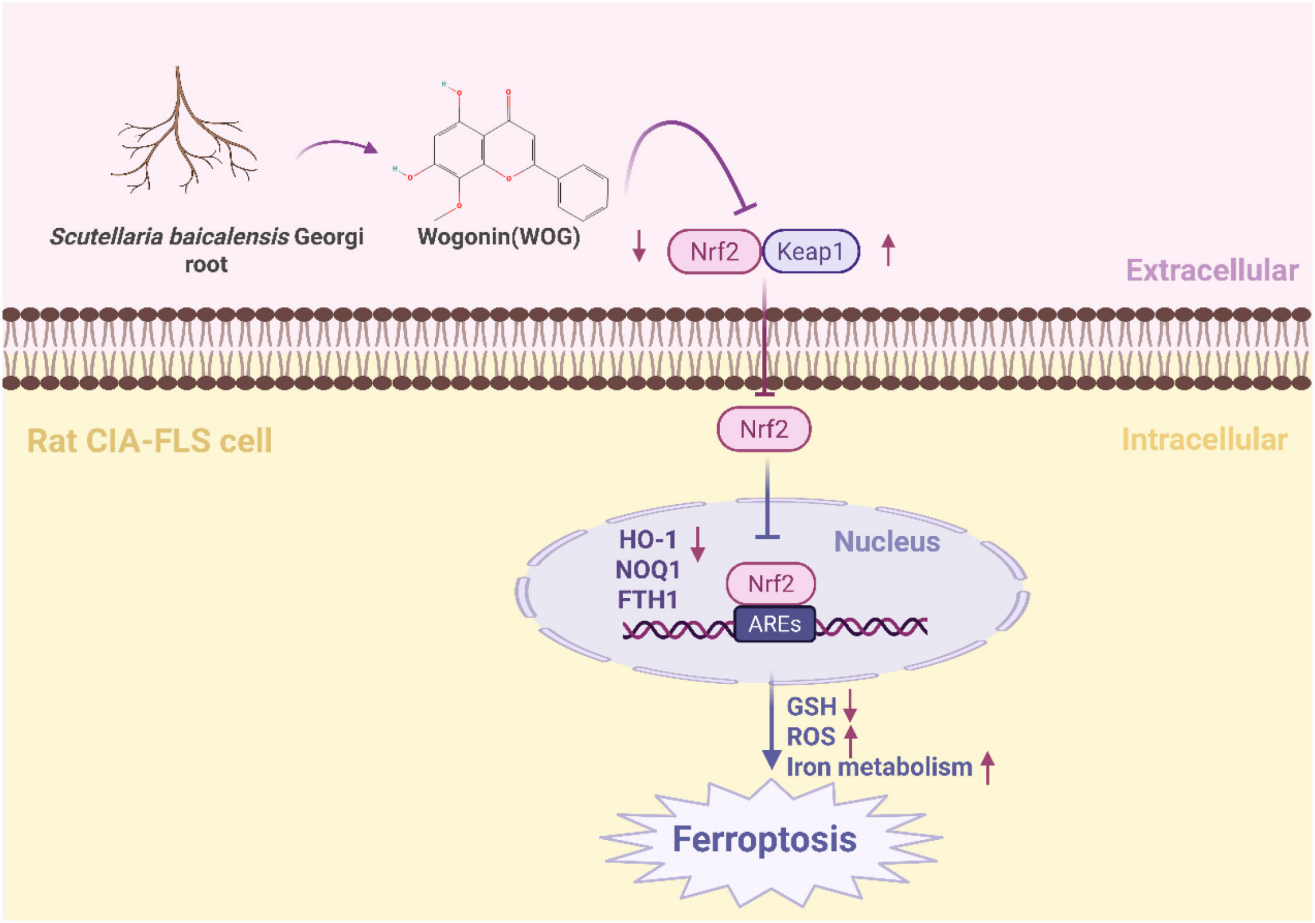
WOG inhibits ferroptosis through the suppression of the Nrf2/HO-1 signaling pathway. Nrf2 forms complexes with other proteins and binds to antioxidant response elements (AREs) in DNA to initiate transcription of various genes including HO-1. HO-1 helps cells to combat oxidative stress and maintain redox homeostasis, which promotes overall cellular health and resilience. WOG promoted the overall health and resilience of the cells through inhibition of the NRF2/HO-1 pathway, Nrf2 and HO-1 protein expression levels were significantly decreased and KEAP1 levels were significantly increased, inducing ferroptosis in rat CIA-FLS cells (44). The figure was created via BioRender (https://BioRender.com).

The Nrf2/HO-1 signaling pathway serves as a crucial regulator of cellular ferroptosis (43) and represents a key defense mechanism against oxidative stress (44). It has been shown that ibuprofen can induce ferroptosis in glioblastoma cells by inhibiting the expression of GPX4 and solute carrier family 7 member 11 (SLC7A11) through inhibition of the nuclear factor erythroid 2-related factor 2 (Nrf2) pathway (45). Wogonin (WOG), a natural flavone isolated from the roots of *Scutellaria baicalensis*, exhibits potent anti-inflammatory and antioxidant properties (46). Notably, it has been shown that WOG can promote oxidative stress through inhibition of the Nrf2/HO-1 pathway, to induce ferroptosis and reducing viability in rat CIA-FLS cells. Specifically, Wogonin treatment resulted in a significant decrease in CIA-FLS cell viability, a significant increase in oxidative stress levels and reactive oxygen species (ROS) content, a significant decrease in Nrf2 and HO-1 protein expression, and a significant increase in KEAP-1 levels (44) (**Figure 1**).

#### 4.1.2 JAK/STAT signaling pathway

**Figure 2:**
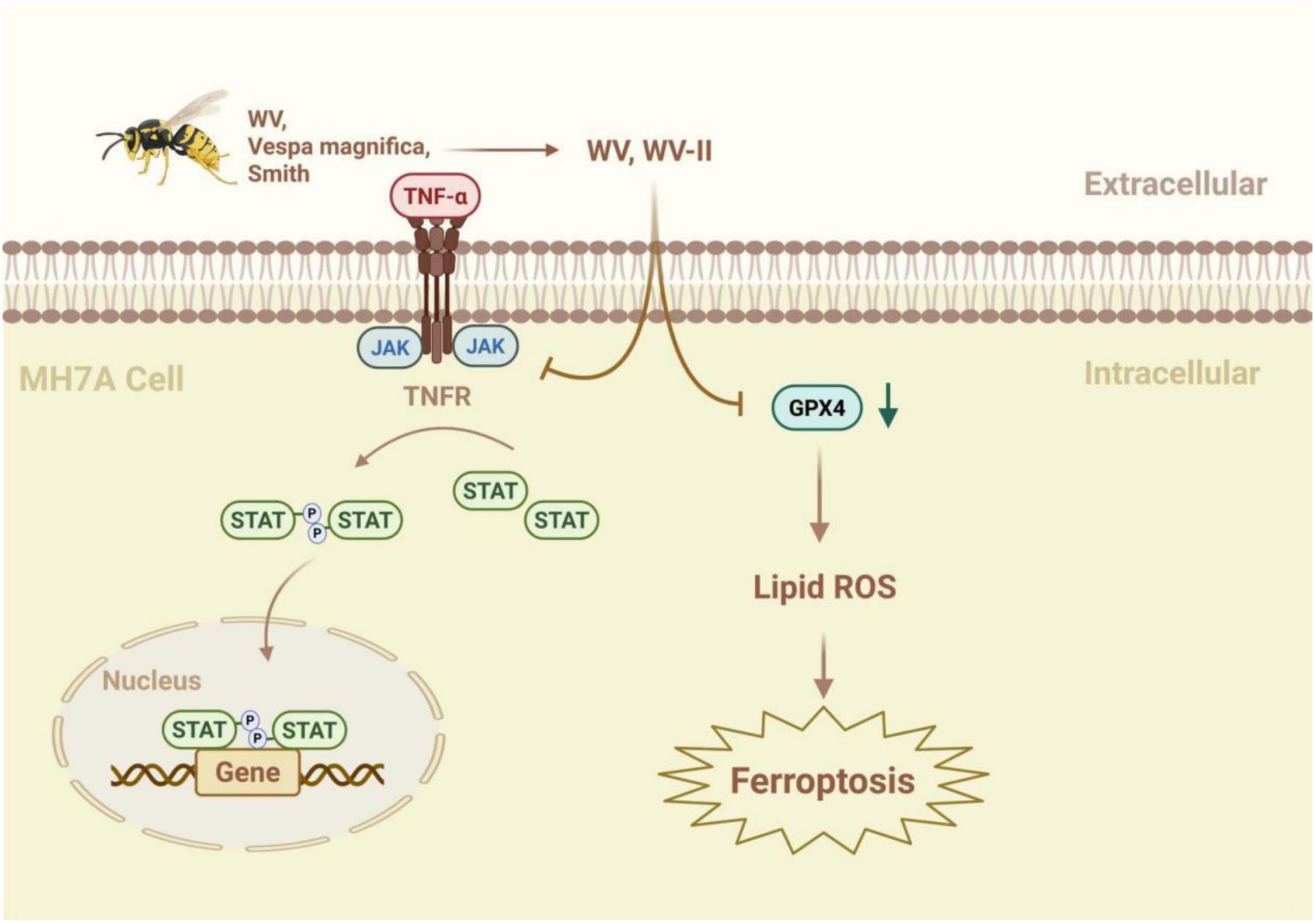
The mechanism by which WV and WV-II activate ferroptosis to inhibit the proliferation of synovial fibroblast-like cells. WV and WV-II decreased the levels of IL-1β and IL-6 in MH7A cells by inhibiting the JAK/STAT signaling pathway. In addition, WV and WV-II accumulated lipid ROS and induced GPX4-mediated ferroptosis, thereby inhibiting inflammation and cell proliferation in MH7A cells (49). The figure was created via BioRender (https://BioRender.com).

The JAK/STAT signaling pathway plays a significant role in various inflammatory diseases (47) and substantially influences lipid peroxidation and iron metabolism through redox system regulation (48). Wasp venom (WV, Vespa magnifica, Smith) exerts anti-inflammatory effects by regulating the JAK/STAT signaling pathway and promotes ferritin deposition in RA treatment (49). In addition, WV and WV-II (molecular weight 3–10 kDa) downregulate TrxR (thioredoxin reductase) activity, generate ROS, induce apoptosis, and accumulate lipid ROS, to induce GPX4-mediated ferroptosis (49). Jolkinolide B (JB), an ent-abietane-type diterpenoid derived from *Euphorbia fischeriana* Steud (known as “lang-du” in TCM), exhibits multiple pharmacological activities, including anti-inflammatory, anticancer, and anti-tuberculosis effects. Studies indicate that JB ameliorates RA by suppressing inflammatory factor expression in collagen-induced arthritis (CIA) rat ankle joints and inhibiting JAK/STAT pathway activation in RAW264.7 cells (50) (**Figure 2**).

#### 4.1.3 GPX4/GSH

**Figure 3:**
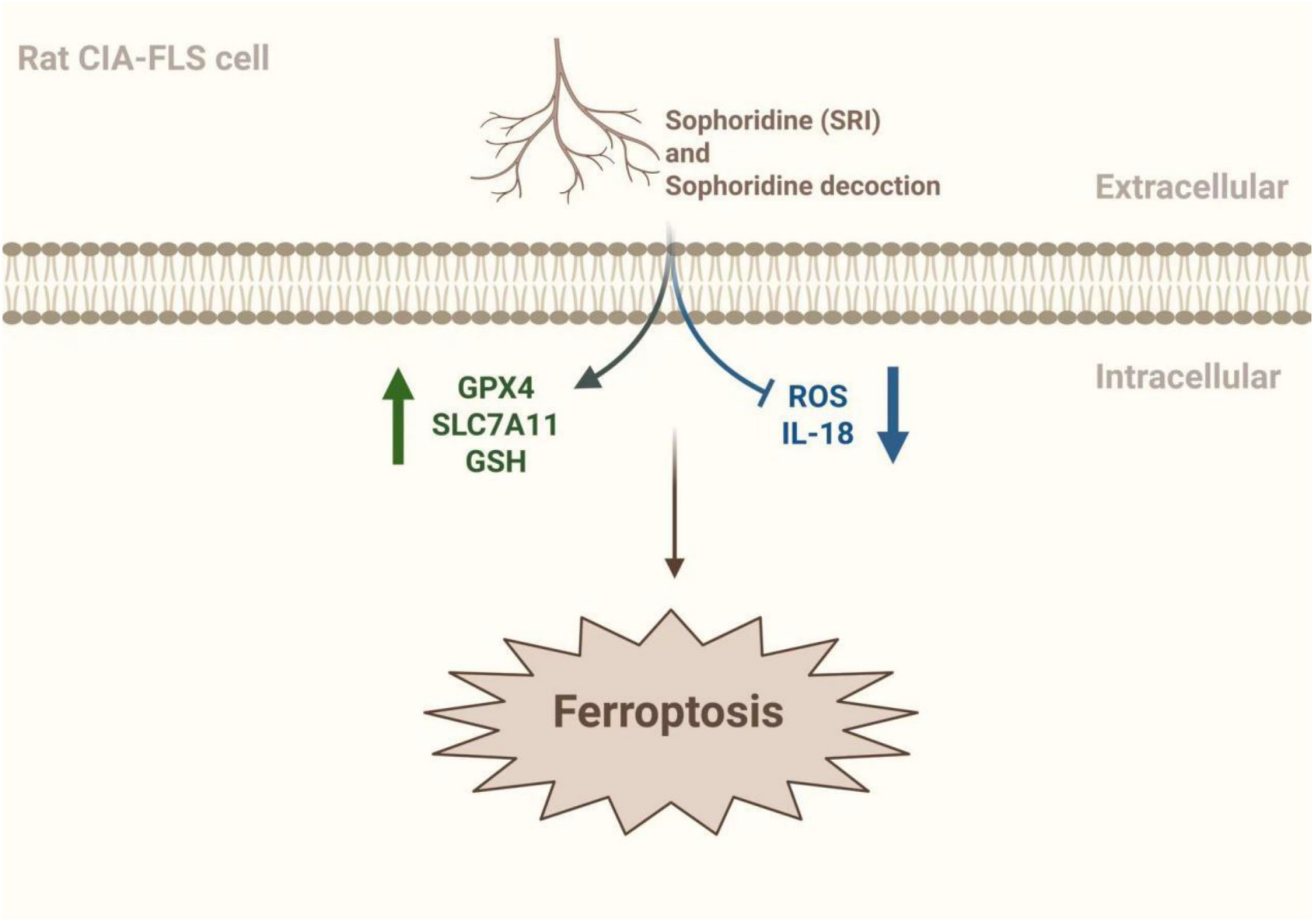
The mechanism by which Sophoridine and Sophoridine regulate ferroptosis. Sophoridine (SRI) and Sophoridine decoction can activate the expression of GSH, GPX4 and SLC7A11 to a certain extent, and inhibit the expression of ROS and IL-18 to a certain extent, and may improve the symptoms of RA by regulating ferroptosis (54). The figure was created via BioRender (https://BioRender.com).

GPX4 is a GSH- and selenium-dependent glutathione peroxidase that plays a crucial role as a central ferroptosis inhibitor in cells (51). Because of its unique catalytic mechanism, it can convert polyunsaturated fatty acid phospholipid hydroperoxides into equivalent phosphatidyl alcohols, inhibiting the production of lipid hydroperoxides and blocking the ferroptosis process (52). Studies have shown that the ROS and lipid peroxides in serum and synovial fluid were elevated in RA patients. In contrast, the expression levels of GSH and glutathione peroxidase in blood were reduced, with a weakened resistance to oxidative stress. In lipopolysaccharide-induced synoviocytes, GPX4 level were reduced and the Xc^-^/SLC7A11/GPX4 axis was significantly inhibited (53). The Chinese herbs Bitter Ginseng Soup and Sophoridine (SRI) have been shown to enhance antioxidant capacity in addition to upregulating GSH and GPX4 expression. It can also downregulate ROS and IL-8 to reduce oxidative damage. Experimental results confirmed that it significantly reduced synovial destruction and joint Fe^3+^ deposition in RA mice. In addition, HE staining showed that Bitter Ginseng Soup reduced synovial cell proliferation and inflammatory cell erosion, and Prussian blue staining showed that Bitter Ginseng Soup reduced Fe^3+^ deposition. Meanwhile, ELISA and IHC showed that Bitter Ginseng Soup and SRI could up-regulate the expression of GSH and GPX4, and down-regulate the levels of ROS and IL-18. These results suggest that Bitter Ginseng Soup and SRI can improve RA symptoms by regulating ferroptosis-related factors (54) (**Figure 3**).

#### 4.1.4 P53/SLC7A11/GPX4

**Figure 4:**
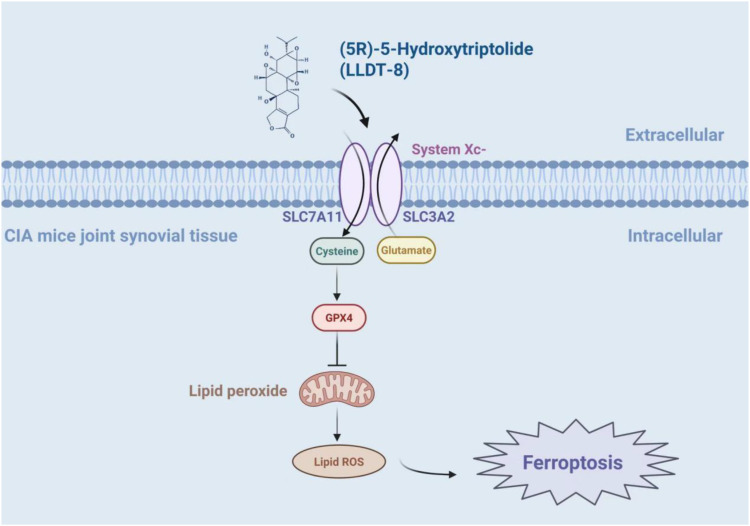
The mechanism of LLDT-8 inhibiting immune-inflammation response in CIA mice. LLDT-8 effectively attenuates synovial hyperplasia and inflammatory infiltration in CIA mice and alleviates joint histopathological damage, and its mechanism may be related to the modulation of the p53/SLC7A11/GPX4 signaling pathway and the increase in ferroptosis sensitivity (56). The figure was created via BioRender (https://BioRender.com).

The tumor suppressor p53 regulates ferroptosis by suppressing SLC7A11 expression, to inhibit cystine uptake (55). (5R)-5-Hydroxytriptolide (LLDT-8, “Lei Teng Shu”), a structurally optimized derivative of triptolide, maintains potent bioactivity while exhibiting reduced toxicity. Notably, LLDT-8 ameliorates joint pathology in CIA mice by modulating the p53/SLC7A11/GPX4 signaling axis, resulting in reduced synovial hyperplasia and inflammatory infiltration. The study used LLDT-8 (0.5 mg/kg) as a pharmacological intervention in CIA-modeled male DBA mice. The results showed that compared with other groups, the LLDT-8 group could significantly improve the swelling and deformation of the joints, reduce the clinical points of arthritis, reduce the bone destruction of the joints, as well as reduce the inflammatory cell infiltration and synovial hyperplasia of the mice, and the expression levels of serum MMP-3, TNF-α, IL-1β, and IL-6 were significantly reduced, and the GSH and NADPH levels of the joint tissues were significantly reduced, and the contents of ROS and MDA were significantly increased, and the expressions of SLC7A11, GPX4 protein and mRNA were significantly increased. SLC7A11, GPX4 protein and mRNA expression were significantly down-regulated, and p53 protein and mRNA expression were up-regulated (56). In addition to this, it has been shown that the inhibitory effect of LLDT-8 on RA FLS is dependent on the WAKMAR2/miR-4478/E2F1/p53 axis (57). Other ways in which it acts may also be by blocking the activation of the NF-κB signaling pathway by inhibiting p65 activity and p65 entry into the nucleus, promoting phosphorylated degradation of IκBα, and inhibiting the expression of its downstream IL-6 and IL-8 in wells, thus blocking the immune-inflammatory response in RA (58) (**Figure 4**) (**Table 1**).

**Table 1:** Single-herb TCM for RA treatment.

Name	Main active ingredients	Chemical formula	Experimental model	Mechanism of action	References
Common Threewingnut Root	LLDT-8*	C_20_H_24_O_7_ 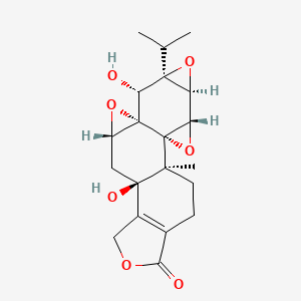	Male DBA mice with RA(CIA) model prepared by type II collagen induction method	Regulation of ferroptosis through the p53/SLC7A11/GPX4 signaling pathway increases ferroptosis sensitivity.	(56)
Cyathula officinalis K.C.Kuan	C. *officinalis* (CES)	C_29_H_44_O_8_ 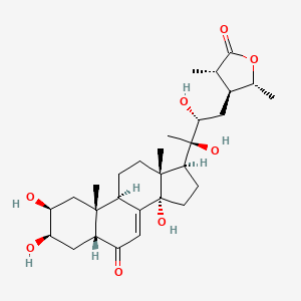	Human synovial cell line MH7A, male Wistar rats	Anti-RA synovial proliferation by inhibiting the expression of inflammatory factors and MMPs, inducing ferroptosis in FLSs, and inhibiting phosphorylation of the PI3K/AKT signaling pathway.	(59)
Elettaria Cardamomum	Cardamonin (CAR)	C_16_H_14_O_4_ 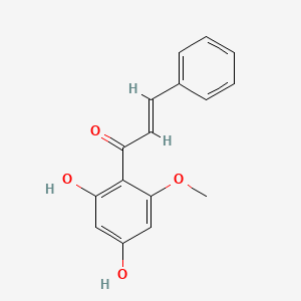	Sixty 6-week-old male wildtype C57BL/6J mice were injected intraperitoneally with iron dextran (500 mg/kg) for iron overload induction.	Prevention of iron overload-induced arthritis by promoting SIRT1 expression and inhibiting the p38MAPK pathway, attenuating ROS production and NLRP3 inflammasome activation.	(13)
*Kadsura heteroclita* (Roxb.) Craib (Xuetong)	Xuetongsu (XTS)	C_30_H_44_O_4_ 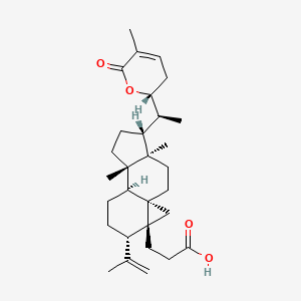	50 male Sprague-Dawley (SD) rats weighing 60–80 g	XTS inhibited LPS-induced inflammation in RAFLS and RAW264.7 cells without causing organ damage by modulating JAK2/STAT3 and NF-κB signaling pathways.	(60)
Langdu	Jolkinolide B (JB)	C_20_H_26_O_4_ 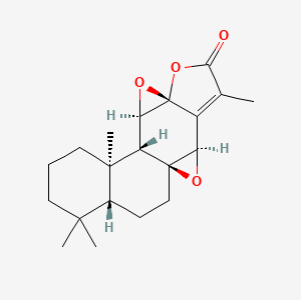	CIA model induction in SPF-grade SD rats (male, 8 weeks, 160 ± 20 g)	Inhibition of mRNA expression of inflammatory factors in the ankle joint of CIA rats and reduction in the concentration of these factors in LPS-induced RAW264.7 macrophages and reduction in the protein expression level of the JAK2/STAT3 pathway.	(50)
Lightyellow sophora root	Sophoridine (SRI)	C_15_H_24_N_2_O 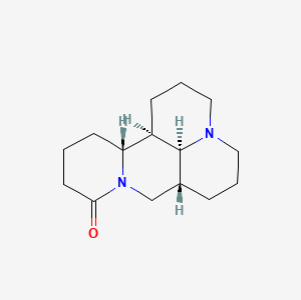	Kunming mice	Activate the expression of GSH, GPX4, and SLC7A11 to a certain extent, and inhibit the expression of ROS and IL-18, which may improve the symptoms of RA by regulating ferroptosis.	(54)
R. officinale Baill	Emodin (Emo)	C_15_H_10_O_5_ 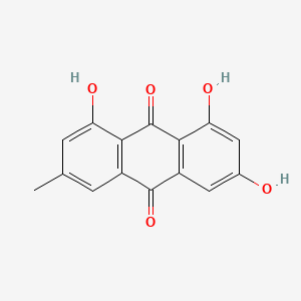	Female SD rats, CIA model prepared by type II collagen induction method	Inhibition of RA bone destruction by regulating ACSL4, SLC7A11, GPX4, FTH1 content and reducing MMP3 and MMP13 expression	(61)
*Scutellaria baicalensis* Georgi	Wogonin (WOG)	C_16_H_12_O_5_ 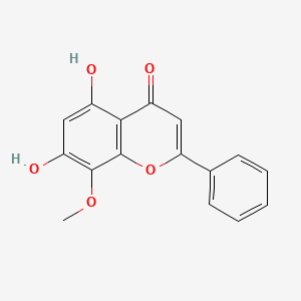	Rat CIA-FLS cells	Promotion of oxidative stress-induced ferroptosis in rat CIA-FLS cells *via* the Nrf2/HO-1 signaling pathway	(44)
Wasp venom (WV, Vespa magnifica, Smith)	WV, WV-II		Human synovial cell lineMH7A, HepG2	By regulating the JAK/STAT signaling pathway, redox homeostasis and ferroptosis in MH7A cells	(49)

*(5R)-5-Hydroxy Lidenolide (LLDT-8) is a new derivative synthesized by modifying the chemical structure of Common Threewingnut Root with Triptolide, the main active ingredient of Common Threewingnut Root, as the lead compound.

### 4.2 Polyherbal formulation

The SLC7A11/GSH/GPX4 signaling pathway is closely linked to ferroptosis (62). Jinwu Jiangu capsule (JWJGC), a traditional Chinese medicine formulation, comprises eight herbal components: Cibotium barometz (L.) J.Sm. (Cibotii Rhizoma), Zaocys dhumnades (Cantor) (Zaocys), Periploca forrestii Schltr. (Periplocae Cortex), Homalomena occulta (Lour.) Schott (Homalomenae Rhizoma), Paeonia lactiflora Pall. (Paeoniae Radix Alba), Panax notoginseng (Burkill) F.H.Chen (Notoginseng Radix et Rhizoma), Curcuma longa L. (Curcumae Longae Rhizoma), and Sinomenium acutum (Thunb.) Rehder & E.H.Wilson (Sinomenii Caulis), demonstrates anti-ferroptosis, in CIA rats. Mechanistically, JWJGC upregulates the GSH/GPX4 axis and modulates the SLC7A11-dependent pathway in M1 macrophages to attenuate inflammatory responses (63).

In addition, other polyherbal formulation such as Gubi Zhitong Formula (GBZTF) and Shaoyao Gancao Decoction can also provide ideas for the treatment of arthritis. Taking osteoarthritis (OA) as an example, GBZTF can inhibit the expression of ferroptosis-related genes (e.g., ETV4, SLC7A11, GPX4) by regulating the serum metabolite α-ketoglutaric acid (α-KG), to alleviate the progression of OA (64). Shaoyao Gancao Decoction has a better improvement effect on the inflammatory immune response and pathological injury of the CIA rat model and has a certain inhibitory effect on IL-1β-induced proliferation and inflammation of MH7A. The mechanism may be related to the modulation of the TNF-α/NF-κB signaling pathway and related inflammatory factors (65) (**Table 2**).

**Table 2:** Polyherbal formulation for RA treatment.

Name	Main active ingredients	Experimental model	Mechanism of action	References
Duhuo Jisheng Decoction	*Angelicae Pubescentis* Radix, *Asarum heterotropoides* F. Schmidt, *Cinnamomum cassia* (L.) D. Don, *Saposhnikovia divaricate* (Turcz.) Schischk., Gentiana, *Eucommia ulmoides* Oliv., *Achyranthes bidentata* Blume, *Taxillus chinensis (DC.) Danser.*, *Rehmanniaglutinosa* (Gaertn.) Libosch. ex Fisch. & C. A. Mey., peony, *Angelica sinensis* (Oliv.) Diels, Ligusticum sinense 'Chuanxiong', *Panax ginseng* C. A. Mey., *Poria cocos (Schw.) Wolf*, *Glycyrrhiza uralensis* Fisch., and 15 other kinds of TCM compositions.	SD rats	Therapeutic effects through modulation of the Wnt/β-catenin signaling pathway, MAPK signaling pathway, NF-κB pathway, JAK/STA signaling pathway, and PI3K/AKT signaling pathway	(14)
Ermiao Powder	*Atractylodes Lancea* (Thunb.) DC., *Phellodendron amurense* Rupr.	weighted gene co-expression network, WGCNA, and molecular dynamics	Reduction of ferroptosis and consequent improvement of RA through modulation of ALOX5 activity in synovial tissue, but lack of ex vivo assays.	(66)
Fengshi Gutong capsule (FSGT)	Aconiti radix cocta (boiled root of Aconitum carmichaelii Debeaux), Aconiti kusnezoffii radix cocta (boiled root of Aconitum kusnezoffii Rchb), Carthami flos (flowers of Carthamus tinctorius L), Glycyrrhizae radix et rhizoma (root and rhizome of Glycyrrhiza uralensis Fisch. ex DC), Chaenomelis fructus (Chaenomelis fructus, fructus of Chaenomeles speciosa [Sweet] Nakai). Mume fructus (fructus of Prunus mume [Siebold] Siebold et Zucc) and Ephedrae herba (rhizome of Ephedra sinica Stapf)	Male SD rats (150–170 g)	FSGT ameliorated CIA-induced RA bone destruction in rats by inhibiting TRAF-6/NFκB/NFATc1 signal.	(67)
Jinwu Jiangu capsule (JWJGC)	Cibotium barometz (L.) J.Sm. (Cibotii Rhizoma), Zaocys dhumnades (Cantor) (Zaocys), Periploca forrestii Schltr. (Periplocae Cortex), Homalomena occulta (Lour.) Schott (Homalomenae Rhizoma), Paeonia lactiflora Pall. (Paeoniae Radix Alba), Panax notoginseng (Burkill) F.H.Chen (Notoginseng Radix et Rhizoma), Curcuma longa L. (Curcumae Longae Rhizoma), and Sinomenium acutum (Thunb.) Rehder & E.H.Wilson (Sinomenii Caulis)	Wistar female rats	Improvement of RA by integrated regulation of the SLC7A11/GSH/GPX4 pathway in M1 macrophages	(63)
SiMiao Wan (SMW)	*Atractylodes Lancea* (Thunb.) DC., *Achyranthes bidentata* Blume, *Coix lacryma-jobi L.var.mayuen (Roman.) Stapf*, Phellodendron Chinese Cortex	Male SD rats	Therapeutic effects on RA-ILD through modulation of lipid and atherosclerosis signaling pathways and TNF and IL-17 signaling pathways	(68)

### 4.3 Traditional therapy

TCM employs a comprehensive therapeutic system incorporating both herbal medicine and non-pharmacological modalities. These traditional interventions, including diet therapy, aromatherapy, acupuncture, moxibustion, massage, cupping, and tai chi, represent the accumulated clinical wisdom of China’s diverse ethnic traditions (69). Among these, acupuncture and moxibustion are the most widely utilized modalities. The two therapies work by stimulating specific surface points on the body (known as “acupoints”) to produce therapeutic effects.

The symptoms of rheumatoid arthritis can be alleviated by acupuncture (an external treatment of traditional Chinese medicine). Acupuncture (including electroacupuncture, warm acupuncture, etc.) has shown significant effects in improving joint function, reducing inflammation and pain in RA patients. Electroacupuncture has the most significant effect in improving MMSE score (SMD 3.66), followed by warm acupuncture (SMD 3.78) and moxibustion (SMD 3.47) (70). For example, acupuncture at Zusanli (ST36) can significantly improve foot swelling, pain and joint inflammatory cell infiltration in collagen-induced arthritis (CIA) mice. The mechanism may be related to the inhibition of pro-inflammatory cytokines (such as TNF-α, IL-6) and the promotion of tissue repair (71). Acupuncture alleviates synovitis in collagen-induced arthritis mice by inhibiting iron phosphorus poisoning through butyric acid (72). In addition, moxibustion regulation of iron lipid metabolism pathway can also improve synovitis inflammation in rheumatoid arthritis rats (73). Clinical selection should be based on the main symptoms of patients: if pain is the main priority, acupuncture can be preferred. If accompanied by obvious iron metabolism disorder or chronic inflammation, moxibustion may be a better complementary therapy (36, 74).

Acupuncture, administered *via* manual needling, electroacupuncture, or transcutaneous electrical acupoint stimulation, modulates inflammatory responses by regulating cytokine production and recruitment at injury sites. M1/M2 macrophage polarization, regulated by this mechanism, enhances anti-inflammatory effects, reduces pain, and promotes tissue repair (75). Moxibustion, which involves burning Artemisia argyi or Artemisia vulgaris at acupoints, exerts anti-inflammatory effects by suppressing mast cell-derived cyclooxygenase (COX), interleukin-6 (IL-6), tumor necrosis factor-α (TNF-α), and other inflammatory mediators. This treatment attenuates cartilage damage and reduces macrophage infiltration (76).

Other therapies such as bee-acupuncture therapy, herbal fumigation and wax therapy, cupping, gua sha, and tuina have also shown potential therapeutic advantages. Studies have shown that bee-acupuncture therapy can effectively relieve clinical symptoms and improve joint conditions in the treatment of early RA patients (77). Hufutongbian scraping therapy can effectively improve pain symptoms and improve the ability to do daily life activities through the stimulation of specific acupoints (78). In addition, non-pharmacological interventions in early RA patients using TCM fumigation combined with acupressure can effectively improve patients’ laboratory indicators and nail fold microcirculation levels without increasing the risk of adverse reactions (79) (**Table 3**).

**Table 3:** Traditional therapies for RA.

Name	Region of action	Experimental model	Mechanism of action	References
Acupuncture	"shenshu" (BL23) and "zusanli" (ST36) acupoints	Specific pathogen-free DBA/1 male mice	Acupuncture at the "shenshu" and "zusanli" points can promote the production of butyric acid and inhibit ferroptosis through the SREBP1/SCD1/GPX4 pathway, to effectively alleviate synovitis in CIA mice.	(72)
Moxibustion	"shenshu" (BL23) and "zusanli" (ST36) acupoints	Male clean-grade SD rats	Improvement of synovial inflammatory injury in RA rats by regulating the ferroptosis-lipid metabolism pathway may be related to the reduction of lipid peroxidation and ROS levels and the inhibition of ferroptosis development. In addition, it can also reduce serum MDA and ROS and increase GSH.	(73)

## 5 Conclusions and perspectives

This review summarizes the role of ferroptosis in RA pathogenesis and examines the therapeutic mechanisms of TCM, including active single-herb TCM, polyherbal formulation, and non-pharmacological therapies. Internal and external therapies of TCM have shown unique advantages and significant potential in RA treatment through multi-target regulation (anti-inflammatory, immune balance, bone protection). While preclinical studies in animal models demonstrate promising results, the clinical efficacy of these TCM approaches requires validation through rigorous randomized controlled trials. Current RA management remains predominantly based on Western medicine, and comparative effectiveness studies are needed to evaluate whether TCM offers comparable or superior outcomes. It is worth noting that when combined with Western drugs (e.g., glucocorticoids), Chinese medicines have been significantly shown to enhance their efficacy, synergize their effects, and help reduce the side effects of Western drugs, such as gastrointestinal reactions and immunosuppression. The growing trend toward integrative medicine underscores the importance of multidisciplinary research to precisely characterize TCM targets while leveraging Western therapeutic advantages. Combination of Chinese and Western medicine can break through the limitations of monotherapy, reduce adverse effects and improve treatment efficacy. For example, combined Chinese and Western medicine treatment can reduce the adverse effects of non-steroidal anti-inflammatory drugs (NSAIDs) and disease-modifying anti-rheumatic drugs (DMARDs), while improving the safety and effectiveness of treatment (80). In addition, the clinical application of combined Chinese and Western medicine treatments in RA, including internal and external treatments of TCM, non-pharmacological therapies such as acupuncture, tuina massage, and a combined Chinese and Western medicine care model, has shown good efficacy.

Currently, TCM is facing several key challenges in the treatment of RA: first, the lack of unified diagnostic and treatment standards and authoritative clinical guidelines, leading to confusion and uncertainty in the choice of treatment for doctors and patients (81); second, the quality of the existing clinical studies is generally poor, mostly small samples or case reports, and the lack of high-quality randomized controlled trials and multicenter studies, which affects the credibility and repeatability of the results (82); third, there is insufficient research on the mechanism of action, and the pharmacological and molecular biological foundations are weak; fourth, the mechanism of action is insufficient research, pharmacology and molecular biology foundation is weak, has not yet formed a scientific and systematic theoretical support; fifth, the therapeutic effect of individual differences is large, a single therapy is difficult to completely control the complex condition. These factors together limit the wide application and recognition of TCM in the field of RA, and the combination of Chinese and Western medicine is regarded as a possible development direction.

In the future, it is important to deepen the research on the relevant mechanisms of TCM and to combine new therapeutic means and methods. This will promote the standardization and precision of combined Chinese and Western medicine protocols, ultimately improving the treatment outcomes for RA patients. With the in-depth study of combined Chinese and Western medicine treatment, the future direction of development includes strengthening the research on the mechanism of combined Chinese and Western medicine treatment, using modern technological means (e.g. artificial intelligence, big data) to assist Chinese medicine treatment, and improving the personalization and precision of treatment. In addition, combined Chinese and Western medicine treatment is also important in the early diagnosis and prevention of RA (83). In addition, identifying key ferroptosis-related molecular targets for TCM intervention has important research value. The in-depth study of the molecular mechanism of ferroptosis can provide a theoretical basis for the development of novel therapeutic strategies based on the regulation of ferroptosis. In the future, the specific mechanism of ferroptosis in RA can be further clarified, the regulatory network of ferroptosis-related molecular targets can be explored, and novel therapeutic strategies based on ferroptosis regulation can be developed. In addition, combining the advantages of TCM and exploring the roles of herbal components in ferroptosis regulation is expected to provide new ideas and methods for the treatment of RA.
